# Occupational stress, burnout and job satisfaction among optometrists in Spain: A cross‐sectional study

**DOI:** 10.1111/opo.70004

**Published:** 2025-08-19

**Authors:** Cristina Alvarez‐Peregrina, Gonzalo Valdes‐Soria, Miguel Ángel Sánchez‐Tena

**Affiliations:** ^1^ Department of Optometry and Vision, Faculty of Optics and Optometry Universidad Complutense de Madrid Madrid Spain; ^2^ Ocupharm Research Group, Faculty of Optics and Optometry Complutense University of Madrid Madrid Spain; ^3^ ISEC LISBOA—Instituto Superior de Educação e Ciências Lisbon Portugal

**Keywords:** burnout, depersonalisation, emotional exhaustion, job satisfaction, occupational stress, optometrist

## Abstract

**Purpose:**

Job satisfaction is an attitudinal variable that describes how people feel in their position. Recent studies indicate that only 45% of Spanish optometrists report job satisfaction, lower than their counterparts in other countries. This study aimed to exhaustively assess factors influencing stress, burnout and job satisfaction within the optometry profession in Spain.

**Methods:**

A cross‐sectional survey was developed comprising three validated questionnaires: Perceived Stress Scale, Maslach Burnout Inventory–Human Services Survey and the Font and Roja Job Satisfaction Questionnaire. The survey targeted all registered optometrists in Spain in 2024.

**Results:**

A total of 2259 responses were collected, representing 12.29% of all registered optometrists and ensuring broad demographic representation. The mean age of the responders was 41.20 ± 10.82 years. Spanish optometrists reported moderate job satisfaction and stress levels. Women experienced significantly higher stress, greater emotional exhaustion and lower job satisfaction than men. Regarding the workplace, optometrists working in shopping mall optical stores reported the highest levels of depersonalisation and emotional exhaustion, along with the lowest sense of personal accomplishment and this was the only setting where optometrists reported job dissatisfaction. University‐employed optometrists reported the lowest levels of burnout and the highest job satisfaction. Greater age and professional experience were positively correlated with increased job satisfaction and personal accomplishment, while negatively correlated with burnout indicators.

**Conclusions:**

Interventions are needed to reduce stress and burnout, particularly among younger and female optometrists. Improving working conditions—especially in shopping mall optical stores—could enhance job satisfaction, reduce early career attrition and promote better patient care through improved professional well‐being.

**Key points**

- Spanish optometrists reported moderate job satisfaction and stress levels, with women experiencing higher stress and emotional exhaustion than men.
- Working in shopping mall optical stores led to higher depersonalisation and emotional exhaustion, lower personal accomplishment and overall job dissatisfaction.
- Job satisfaction and personal accomplishment increased with age and years of professional experience.

## INTRODUCTION

Optometrists are the primary healthcare practitioners responsible for providing comprehensive eye and vision care. Their role covers a wide range of tasks including refraction, contact lens fitting, dispensing of prescription spectacles, detection of disease in the eye and rehabilitation of conditions of the visual system.^1^ In Spain, the profession is regulated by law and spans various settings, such as private clinics, optical healthcare establishments, hospitals and universities, offering diverse career opportunities. It is coordinated by the General Council of Optician‐Optometrists of Spain (CGCOO, by its acronym in Spanish), the national governing body that represents and regulates the practice of the optometric profession across the country, promoting professional standards, education and public health initiatives related to visual care.

The World Council of Optometry (WCO) classifies the profession in Spain as Category 3 within a four‐tier competency model, meaning that Spanish optometrists have one of the highest scopes of practice worldwide. This includes optical technology services, visual function services and ocular diagnostic services, such as investigation, examination and evaluation of the eye and adnexa and associated systemic factors, to detect, diagnose and manage disease and additional clinical tests. However, their scope of practice does not include the use of diagnostic drugs, unlike countries such as Finland, Norway, Sweden, Ireland or the UK.^2^

Despite these advanced competencies, recent studies have shown that only 45% of Spanish optometrists report job satisfaction, with salary, career development opportunities and prestige in society as the main reasons for this discontent.^3^ Job satisfaction is an attitudinal variable that describes how people feel about their roles in their position, referring to the positive emotions and sense of fulfilment that employees feel toward their role.^4^, ^5^ It is influenced by factors such as job stability, opportunities for career growth, professional development and work–life balance.^6^ Job dissatisfaction has been related to burnout,^7^ a mental condition that arises when coping strategies that individuals typically utilise to manage stress at work fail. The most widely recognised definition of burnout was proposed by Maslach et al.^8^ as a psychological syndrome marked by emotional exhaustion, depersonalisation and a diminished sense of personal accomplishment, typically occurring in individuals engaged in professional relationships with other people. Health professionals, including optometrists, often face high levels of stress due to heavy workloads, role ambiguity and patient care responsibilities. This accumulation of stress can exhaust individuals to the point where their resources are insufficient to overcome the pressure of a situation,^9^ which can lead to job dissatisfaction, absenteeism and higher intention to leave their profession prematurely.^10^

Several studies underscore the need for more research on interventions and prevention strategies that address the most significant workplace stressors among healthcare practitioners, such as workload, stress and work–life balance.^11^, ^12^, ^13^ The optometric profession in Spain faces significant future challenges and uncertainties. Despite the growing demand for eye care services, optometrists often grapple with issues such as limited recognition of their expertise and inadequate opportunities for professional development.^3^ Moreover, economic pressures and the dual nature of the role—balancing healthcare responsibilities with commercial aspects in optical retail—add complexity to the profession.^14^

Given the increasing demand for optometric services and the relationship between excessive job stress—which can lead to burnout—and burnout itself, that has been reported to correlate with job satisfaction,^7^, ^15^ this study aimed to exhaustively evaluate key factors affecting the optometry profession in Spain with regard to stress, burnout syndrome and job satisfaction using three validated questionnaires.

## METHODS

A cross‐sectional and observational survey was conducted among all registered optometrists listed in the CGCOO at the time of conducting this study (from January to April 2024), including optometrists working at ophthalmological clinics, public and private hospitals, street‐level and shopping mall optical healthcare establishments (including independent, chains and franchise optical centres), as well as optometrists working for research and teaching at universities.

An online questionnaire was developed and shared with all potential participants. The survey was available for a 3‐month period, allowing respondents to complete it at their own pace with no time restrictions. It comprised three sections, each one evaluating a different variable: stress, burnout and job satisfaction. The full questionnaire is available upon reasonable request to the corresponding author.

- *Section 1. Perceived Stress Scale (PSS)*. A widely used 14‐item psychological questionnaire designed to measure the degree to which individuals perceive situations in their lives as stressful over the past month. The responses are also scored on a 5‐point Likert scale (0‐never, 1‐hardly ever, 2‐sometimes, 3‐often, 4‐frequently), with higher scores indicating greater perceived stress. The total score ranges from 0 to 54.^16^, ^17^
- *Section 2. Maslach Burnout Inventory–Human Services Survey (MBI–HSS)*. This questionnaire is based on the original MBI survey designed by Maslach and Jackson^18^ and has been adapted to assess burnout specifically in the human services and healthcare fields. This 22‐item survey assesses three key dimensions of burnout: emotional exhaustion, depersonalisation and personal accomplishment using a series of statements rated on a 5‐point Likert scale (1‐never, 2‐few times a year, 3‐few times a month, 4‐few times a week, 5‐daily). Scores range from 9 to 45 for emotional exhaustion, from 5 to 25 for depersonalisation and from 8 to 40 for personal accomplishment. The MBI–HSS has been recognised as the most valid and reliable multidimensional tool for assessing burnout in human service professionals.^19^, ^20^
- *Section 3. Font–Roja Job Satisfaction Questionnaire*. The 24‐item Spanish language tool developed by Aranaz in 1998^21^ for healthcare professionals determines job satisfaction according to the overall average satisfaction (OAS) score and classifies the responses as satisfied if OAS >0, unsatisfied if OAS <0 and indifferent if OAS = 0. Items are scored on a 5‐point Likert scale ranging from 0 (‘Totally disagree’) to 4 (‘Totally agree’).

Furthermore, comprehensive demographic data was gathered from the participants, including their age, gender, workplace and region of the country where they were located to ensure a representative sample of the studied population.

Statistical analysis was performed using SPSS Statistics, version 29.0.2 (IBM SPSS Statistics, ibm.com). Normal distribution of variables was assessed by the Kolmogorov–Smirnov and Shapiro–Wilks tests. As none of the variables followed a normal distribution, Wilcoxon Mann– Whitney, Kruskal–Wallis and Friedman non‐parametric tests were used to compare the results of the different sections of the survey (stress, burnout and job satisfaction scores) across the different types of working environments (ophthalmological clinics and hospitals, street‐level optical establishments, shopping mall optical stores, universities and others) and gender. Correlations between variables and demographic data were analysed using Spearman's rank correlation coefficient. Statistical significance was set at a 95% confidence interval or *p*‐value <0.05.

## RESULTS

The survey was launched on 26 January, 2024, targeting all registered optometrists in Spain and was available for 3 months. At that time, there were 18,380 registered optometrists in the country, from whom a total of 2259 responses were collected, representing 12.29% of all registered optometrists. The answers were spread across all Spanish regions, ensuring both geographical and demographic diversity that accurately reflected the reality of the sector. Comprehensive demographic data is presented in Table 1.

**TABLE 1:** Descriptive statistics of the study population in terms of age and working experience (mean ± SD).

Socio‐demographic variables	*n* (%)	Age (years)	Experience (years)
All participants	2259 (100)	41.20 ± 10.82	17.80 ± 10.55
Gender			
Men	588 (26.03)	43.89 ± 11.40	19.98 ± 11.25
Women	1673 (74.06)	40.25 ± 10.45	17.03 ± 10.19
Workplace			
Ophthalmological clinics	297 (13.15)	38.54 ± 10.08	15.46 ± 9.50
Street‐level optical establishments	1633 (72.29)	42.14 ± 10.90	18.72 ± 10.72
Shopping mall optical stores	253 (11.20)	38.22 ± 9.70	14.54 ± 9.17
Universities	37 (1.64)	41.38 ± 13.07	18.81 ± 12.73
Other	41 (1.81)	40.93 ± 11.09	17.22 ± 10.81

The mean age of the responders was 41.20 ± 10.82 years. The majority of the participants were women (*n* = 1673; 74.06%) and worked in street‐level optical healthcare establishments (*n* = 1633; 72.29%). There were significant differences in age and working experience between genders, with women being significantly younger and having fewer years of working experience than men (*p* < 0.001; Wilcoxon test). Between working environments, younger people with less working experience were mainly employed in ophthalmological clinics and hospitals or in shopping malls (*p* < 0.001; Kruskal–Wallis test, pairwise comparisons).

### Perceived Stress Scale (PSS)

Stress levels, as measured by the PSS questionnaire and categorised by gender and workplace, are presented in Table 2. Spanish optometrists reported moderate stress levels, with a mean score of 25.86 ± 9.86 (range: 0–54). Women exhibited significantly higher stress than men (*p* = 0.02; Wilcoxon test), while no significant differences were observed concerning the workplace (*p* = 0.12; Kruskal–Wallis test). Additionally, no significant correlation was found between stress and either age (ρ = −0.02; *p* = 0.29; Spearman test) or years of professional experience (ρ = −0.001; *p* = 0.97; Spearman test).

**TABLE 2:** Perceived Stress Scale (PSS) scores (mean ± SD).

PSS	Stress
All participants	25.86 ± 9.86
Gender	
Female	26.11 ± 9.87
Male	25.15 ± 9.80
Workplace	
Ophthalmological clinics	25.74 ± 9.93
Street‐level optical establishments	26.12 ± 9.88
Shopping mall optical stores	24.28 ± 9.50
Universities	25.76 ± 9.77
Others	26.44 ± 10.20

*Note*: Score range from 0 to 54.

### Maslach Burnout Inventory–Human Services Survey (MBI–HSS)

The results for the three burnout variables assessed in the MBI–HSS questionnaire are presented in Table 3. Range‐corrected analysis revealed significant differences among the three dimensions (*p* < 0.001; Friedman test). Personal accomplishment scores were significantly higher than those for emotional exhaustion (*p* < 0.001; Wilcoxon test) and depersonalisation (*p* < 0.001; Wilcoxon test), while depersonalisation was significantly lower than emotional exhaustion (*p* < 0.001; Wilcoxon test).

**TABLE 3:** Maslach Burnout Inventory–Human Services Survey (MBI–HSS) questionnaire scores (mean ± SD).

MBI–HSS	Emotional exhaustion	Depersonalisation	Personal accomplishment
All participants	27.95 ± 9.49	10.48 ± 4.16	30.77 ± 5.86
Gender			
Female	28.40 ± 9.34	10.39 ± 4.10	30.69 ± 5.85
Male	26.71 ± 9.78	10.71 ± 4.33	30.99 ± 5.91
Workplace			
Ophthalmological clinics	26.72 ± 9.11	10.02 ± 3.73	31.36 ± 5.24
Street‐level optical establishments	27.90 ± 9.53	10.37 ± 4.12	31.00 ± 5.86
Shopping mall optical stores	30.77 ± 8.88	11.87 ± 4.42	28.78 ± 5.97
Universities	21.73 ± 8.12	8.51 ± 3.74	31.11 ± 4.61
Others	27.92 ± 10.68	11.50 ± 5.48	29.24 ± 8.04

*Note*: Score ranged from 9 to 45 for emotional exhaustion, from 5 to 25 for depersonalisation and from 8 to 40 for personal accomplishment.

Gender‐based comparisons revealed that emotional exhaustion was significantly higher in women than in men (*p* < 0.001; Wilcoxon test), while no significant differences were found between genders regarding depersonalisation (*p* = 0.20; Wilcoxon test) and personal accomplishment (*p* = 0.24; Wilcoxon test).

According to workplace differences, optometrists working at universities had significantly lower emotional exhaustion compared to those working at street‐level optical healthcare establishments (*p* = 0.002; Kruskal–Wallis, pairwise comparisons), shopping mall optical stores (*p* < 0.001; Kruskal–Wallis, pairwise comparisons), ophthalmological clinics and hospitals (*p* = 0.04; Kruskal–Wallis, pairwise comparisons) or other workplaces (*p* = 0.007; Kruskal–Wallis, pairwise comparisons). Additionally, optometrists working at shopping malls reported significantly higher emotional exhaustion scores than those in ophthalmological or street‐level optical healthcare establishments (*p* < 0.001; Kruskal–Wallis, pairwise comparisons). A similar trend was observed in depersonalisation scores, with university‐employed optometrists reporting the lowest scores compared to all other workplaces (*p* < 0.01; Kruskal–Wallis, pairwise comparisons). Conversely, shopping malls had the highest depersonalisation scores, significantly greater than those observed at ophthalmological and street‐level optical healthcare establishments (*p* < 0.001; Kruskal–Wallis, pairwise comparisons). For personal accomplishment, optometrists working in shopping malls reported significantly lower scores compared to those in street‐level centres and ophthalmological clinics (*p* < 0.001; Kruskal–Wallis, pairwise comparisons), with no significant differences among other comparisons (all *p* > 0.05; Kruskal–Wallis, pairwise comparisons).

Correlation analysis showed a strong negative relationship between emotional exhaustion and depersonalisation with both age (ρ = −0.23 and ρ = −0.21, respectively; *p* < 0.001; Spearman test) and working experience (ρ = −0.22 and ρ = −0.20, respectively; *p* < 0.001; Spearman test). In contrast, personal accomplishment showed a positive correlation with age (ρ = 0.18; *p* < 0.001; Spearman test) and years of working experience (ρ = 0.18; *p* < 0.001; Spearman test).

### Font–Roja job satisfaction questionnaire

Job satisfaction scores, as measured by the Font–Roja questionnaire, are presented in Table 4, categorised by gender and workplace. Spanish optometrists reported overall neutrality regarding job satisfaction, with an OAS score close to zero (0.063 ± 0.514). Significant differences were observed between genders, with men reporting significantly higher job satisfaction than women (*p* < 0.001; Wilcoxon test).

**TABLE 4:** Job Satisfaction scores from the Font–Roja Questionnaire (mean ± SD).

Font–Roja	Overall average satisfaction
All participants	0.063 ± 0.514
Gender	
Female	0.027 ± 0.502
Male	0.164 ± 0.533
Workplace	
Ophthalmological clinics	0.048 ± 0.495
Street‐level optical establishments	0.093 ± 0.512
Shopping mall optical stores	−0.148 ± 0.480
Universities	0.357 ± 0.451
Others	0.052 ± 0.621

*Note*: Score ranged from −2 to +2.

The Font–Roja questionnaire further revealed that optometrists working at shopping malls were the only group with a negative OAS value of −0.148 ± 0.480, meaning dissatisfaction with their jobs. Their score was significantly worse than the job satisfaction of optometrists working at street‐level optical healthcare establishments, ophthalmological clinics and universities (*p* < 0.001; Kruskal–Wallis, pairwise comparisons). University‐employed optometrists reported the highest job satisfaction compared to those working at street‐level optical establishments (*p* = 0.001; Kruskal–Wallis, pairwise comparisons), ophthalmological centres (*p* = 0.01; Kruskal–Wallis, pairwise comparisons) and other workplaces (*p* = 0.003; Kruskal–Wallis, pairwise comparisons). Additionally, the Font–Roja job satisfaction OAS score exhibited a positive correlation with age (ρ = 0.11; *p* < 0.001; Spearman test) and years of working experience (ρ = 0.11; *p* < 0.001; Spearman test).

## DISCUSSION

This study is the first to administer a large‐scale observational survey to Spanish optometrists, integrating three validated questionnaires that assessed key occupational variables: stress, burnout and job satisfaction. Furthermore, the sample is highly representative of the Spanish optometrist population, achieving a 12.29% response rate among all registered optometrists in the country while ensuring comprehensive demographic coverage. The results of the present study may explain why a similar survey recently found that job satisfaction among Spanish optometrists was lower compared with their counterparts in other countries.^3^ This highlights the need for a better understanding of the challenges faced by optometrists in Spain as a way to develop strategies aimed at improving their professional well‐being. The present findings indicate that younger age, limited work experience, female gender and employment in shopping mall settings are significantly associated with lower job satisfaction, burnout and stress, which in turn can affect patient care, increase pressure and limit the practitioners' autonomy.^22^, ^23^

Several studies have assessed job satisfaction and stress of different healthcare professionals, with results varying based on promotion opportunities, working conditions, relationship with co‐workers, the nature of the work, monotony, educational level and country of employment.^22^, ^24^ For example, among Ethiopian healthcare workers, job dissatisfaction, sleeping disorders, longer working hours, female gender and younger age were identified as predisposal factors for job‐related anxiety and occupational stress.^25^ Similar outcomes have been reported in nurses of Latin American countries.^22^ In the United States, the prevalence of depression in the healthcare industry was estimated at 14.8%,^26^ with 46% of healthcare workers reporting burnout in 2022, according to the Centers for Disease Control and Prevention (CDC).^27^ Furthermore, the COVID‐19 pandemic led to a global increase in the prevalence of mental health disorders among healthcare workers, with persistent distress, anxiety and depression,^28^, ^29^ which translated into a significant rise in the prevalence of self‐reported feelings of burnout.^27^ A similar trend was observed in Europe, with burnout rates of 53.2%, 33% and 47.8% for emotional exhaustion, depersonalisation and reduced personal accomplishment, respectively,^27^ and the highest likelihood of exposure to mental health risks for Northern countries followed by those in Western Europe.^29^

In the context of optometrists, a recently published study involving optometrists in the United States reported that 53.6% of responders had experienced burnout symptoms, with a higher prevalence in women than men and the lowest rates observed among those working in academic settings.^30^ These outcomes are closely aligned with the results reported in the present study involving Spanish optometrists. Moreover, Australian practitioners reported higher levels of mental health conditions and burnout compared with the general population and other healthcare professionals, with younger age and burnout being significant risk factors for psychological distress.^31^ This also aligns with the current findings involving Spanish optometrists, where a strong negative correlation exists between emotional exhaustion and depersonalisation with both age and experience. Younger optometrists are more prone to burnout syndrome and experience lower personal accomplishment and job satisfaction. This may be attributed to a lack of resilience, which has been observed among younger optometrists in Asia‐Pacific countries as well as the UK and USA, a key enabler for healthcare personnel to overcome adversities and to develop strategies for academic and professional success.^32^

Excessive job stress is a well‐documented contributor to burnout.^15^ Spanish optometrists reported moderate stress levels at work, similar to Australian optometrists, who identify workload demands and management tasks as primary stressors.^33^ This aligns with findings that Spanish optometrists working in shopping malls experience significantly higher levels of emotional exhaustion and depersonalisation, describing a lack of personal accomplishment and dissatisfaction at their workplace. This is likely due to increased management demands, commercial pressures and extended opening hours in this work environment. In contrast, optometrists working in university settings reported the lowest levels of emotional exhaustion and depersonalisation, possibly due to higher social recognition as university professors and greater job satisfaction. The same outcome has been described among optometrists in the USA,^30^ and similar trends have also been observed among nurses, where perceptions of job status vary across different healthcare institutions.^34^ One key reason underlying these outcomes may be the detrimental work–life balance faced by optometrists working in shopping mall stores. One of the major concerns of optical healthcare establishments in Spain may be their hourly schedule. While street‐level optical establishments and private clinics typically close between 20:00 and 21:00 h, Monday through Friday and are only open on Saturday mornings, optical stores located in shopping malls follow a much longer schedule, usually being open from 10:00 to 22:00 h, Monday to Sunday. This extended work schedule directly impacts employees' quality of life and work–family balance and may also contribute to lower job satisfaction; there by influencing the quality of service, staff retention and having a direct impact on productivity.^35^ Previous research has established a strong connection between work–life balance, intention to leave the profession, staff retention and overall well‐being,^36^ which highlights the critical role of workplace environment and conditions in shaping occupational stress and burnout.

Conversely, studies using similar validated questionnaires among optometrists and vision technicians in Ghana and India report higher job satisfaction levels, with salary, work–life balance and continuing education opportunities being significant contributing factors.^37^, ^38^ Research indicates that optometrists actively set boundaries to mitigate work‐related stress and health issues, developing strategies to manage stress and foster a sense of coherence, particularly when working in remote and rural areas.^39^, ^40^

Regarding gender, Spanish female optometrists reported higher stress and emotional exhaustion than their male counterparts. Several factors may contribute to this disparity, including the uneven distribution of family and domestic responsibilities that has been observed among female physicians in Spain.^41^ Women in the healthcare sector spent more than twice the time performing household duties and were over three times more likely to live alone with children than men. Despite this, both genders shared similar professional workloads, which hindered women's development of leadership activities such as managing, supervising residents, publishing original articles or being a principal investigator.^41^ This gender imbalance has also been observed in the authorship analysis of the five leading optometry journals, where women are under‐represented in senior and leadership positions.^42^ Furthermore, this disparity could also be attributed to the persistent wage gap, with women optometrists in Spain earning 8% less than men in the same professional categories and men occupying higher positions in optical companies and healthcare establishments.^43^ In the USA, although the gender gap has narrowed in recent years, female optometrists still earned 14% less than their male counterparts in 2021,^44^ a pattern also seen among ophthalmologists, with women earning 12.5% less than men during their first year of clinical practice.^45^ Despite women comprising more than double the number of men in healthcare professionals in Spain, gender equity is far from being reached and there is still plenty of room for improvement in the optometric profession.

The results of the present study are consistent with those reported among various other healthcare professionals in Spain, including dermatologists,^46^ emergency medical personnel,^47^, ^48^ nurses^49^, ^50^ and speech‐language pathologists.^51^ These similarities suggest that the challenges identified in stress, burnout and job satisfaction may be widespread across different healthcare fields, rather than being unique to optometry. Regarding burnout dimensions, overall burnout levels among healthcare workers in Spain 1 year after the COVID‐19 pandemic have shown partial recovery. However, there has been an increase in depersonalisation and a decline in self‐fulfilment.^52^

The current findings showed that optometrists exhibit higher levels of emotional exhaustion and depersonalisation compared with other healthcare professionals, while their level of personal accomplishment was comparable to that observed in a previous study involving nurses, physicians, nursing assistants and orderlies in Spain.^53^ Notably, burnout syndrome has also been reported in 66% of ophthalmologists in Spain, Argentina and Mexico, with younger age and public activity being identified as significant predisposal factors,^54^ as well as in 53.6% of US optometrists.^30^ This pattern is consistent with the present findings on Spanish optometrists, suggesting that burnout levels are relatively uniform across the field of vision sciences. Regarding job satisfaction, nurses in Spain reported a medium‐high satisfaction level (67.44% on a 0–100 scale),^34^, ^55^ which was slightly higher than that of Spanish optometrists. The association between face‐to‐face public activity and increased burnout, stress and lower job satisfaction is particularly concerning, given the rising incidence of violence against healthcare personnel, which has become a growing public health issue in Spain.^56^

In view of these findings, interventions are needed to address these challenges on healthcare workers, particularly for younger optometrists. Such interventions may include workplace adjustments and strategies to enhance resilience, promote mental well‐being and ensure patient safety. The relatively high occurrence of mental health issues and burnout among optometry students underscores the need to equip future optometrists with essential skills to support their mental well‐being. For all practicing optometrists, reducing stress factors and improving employment standards, especially in shopping malls, may help prevent burnout and early retirement, thereby retaining talent, attracting new professionals and improving patient care through enhanced job satisfaction.

The limitations of the study, the cross‐sectional and non‐experimental nature of the survey, present some challenges in interpreting the results. Although burnout and stress seem to be related to deteriorating health‐related quality of life and job dissatisfaction, only associations could be suggested, as the observational nature of the study prevented the establishment of cause‐effect relationships. Additionally, while the sample size ensured full demographic coverage and was proportional to the Spanish optometric population, homogeneity in terms of workplace was not achieved. This limitation is particularly relevant considering that 72% of the answers came from street‐level optometrists, whereas only 1.6% came from university‐employed practitioners.

In conclusion, this study found that Spanish optometrists reported moderate job satisfaction, with women experiencing higher stress, greater emotional exhaustion and significantly lower job satisfaction than men. Among workplaces, shopping mall optical stores had the highest levels of depersonalisation and emotional exhaustion, the lowest sense of personal accomplishment and were the only setting where optometrists reported job dissatisfaction. In contrast, university‐employed optometrists reported the lowest levels of burnout and the highest job satisfaction, likely due to greater social recognition and better work–life balance, particularly compared with those in shopping mall stores.

## AUTHOR CONTRIBUTIONS

**Cristina Alvarez‐Peregrina:** Conceptualization (equal); data curation (equal); investigation (equal); methodology (equal); project administration (equal); supervision (equal); validation (equal); writing – review and editing (equal). **Gonzalo Valdes‐Soria:** Formal analysis (equal); writing – original draft (equal). **Miguel Ángel Sánchez‐Tena:** Data curation (equal); investigation (equal); methodology (equal); supervision (equal); validation (equal); visualization (equal); writing – review and editing (equal).

## FUNDING INFORMATION

The Spanish General Council of Opticians and Optometrists funded this study.

## CONFLICT OF INTEREST STATEMENT

The authors declare no competing interests.
